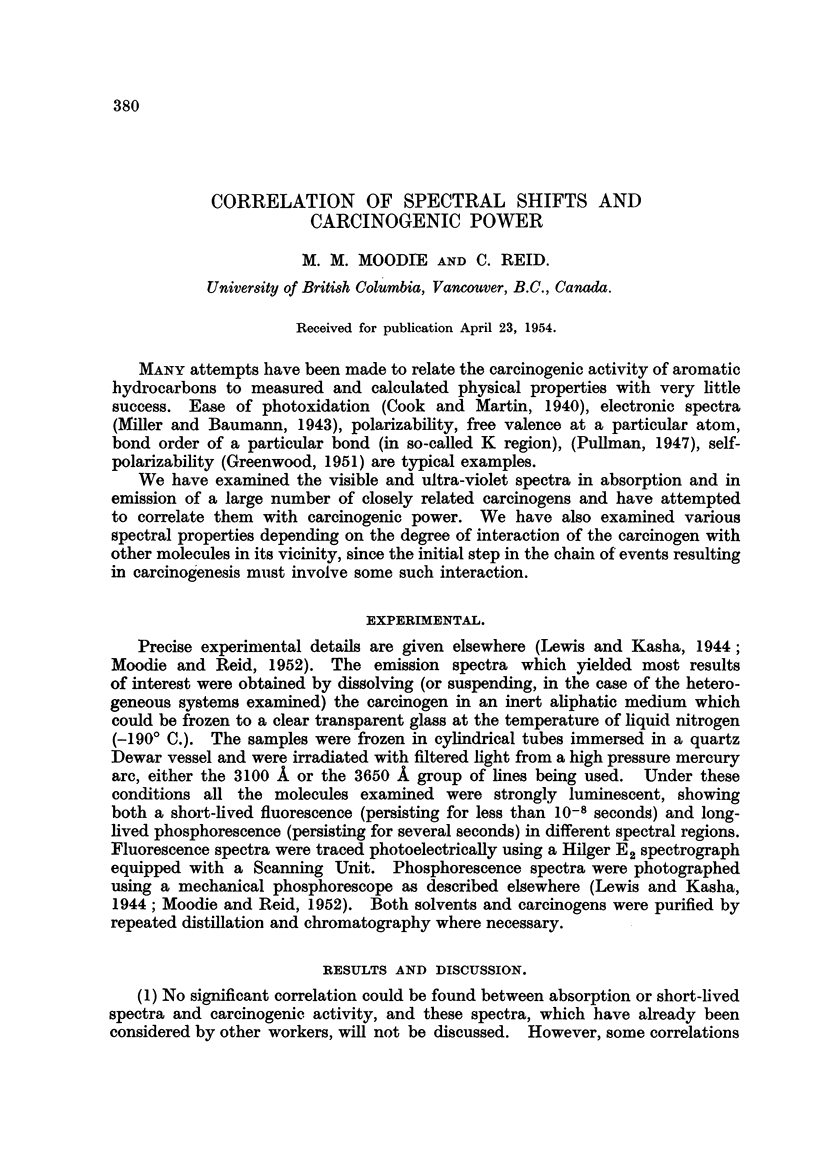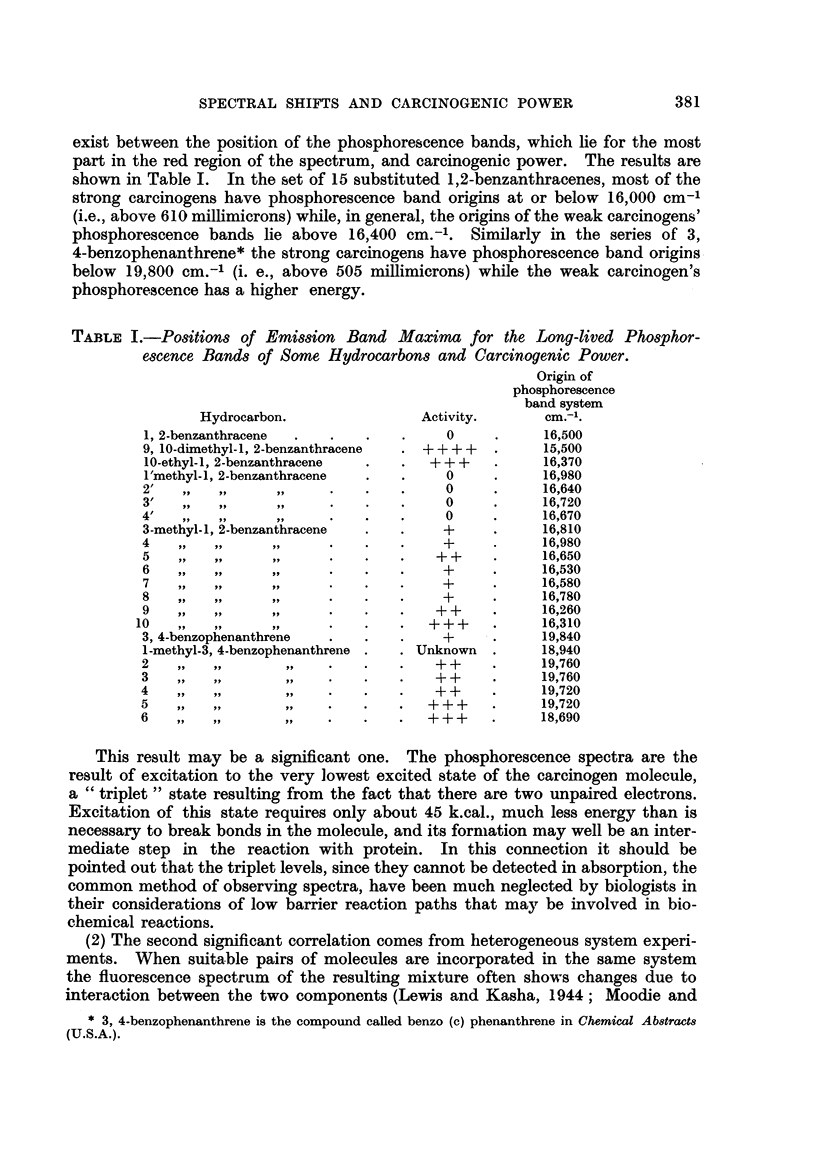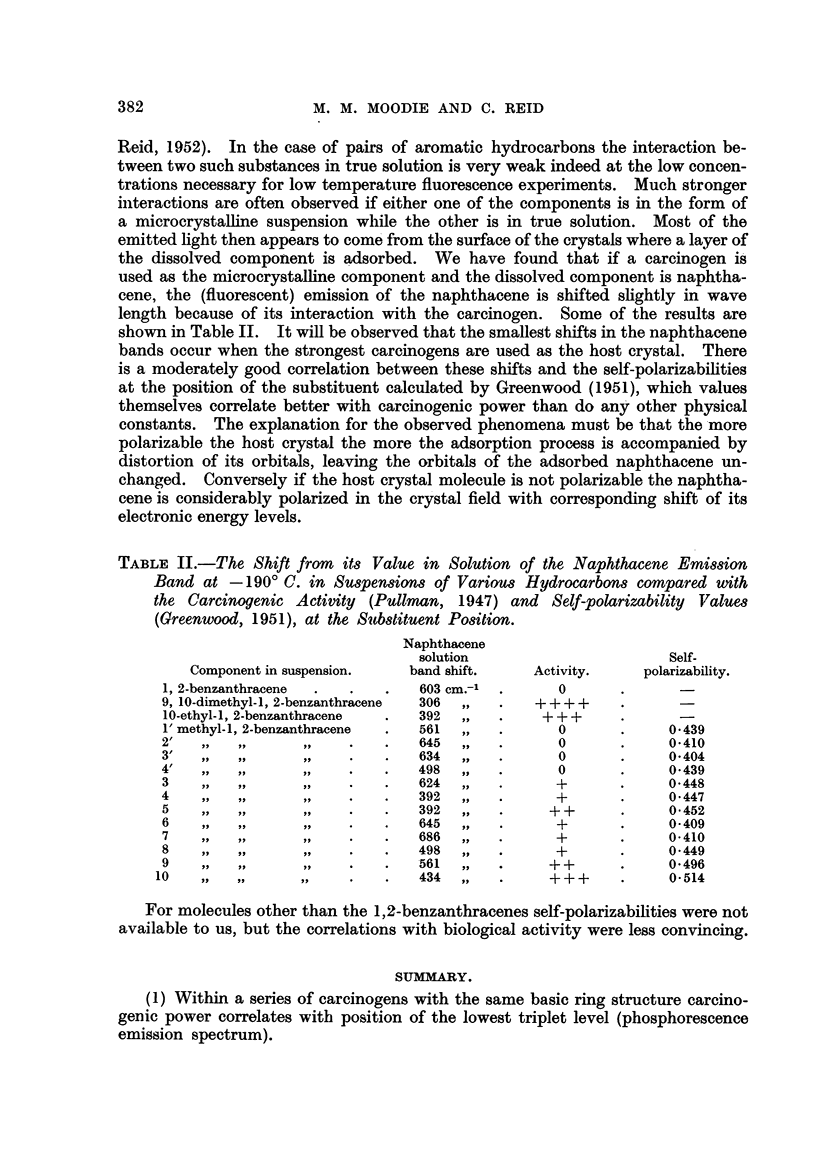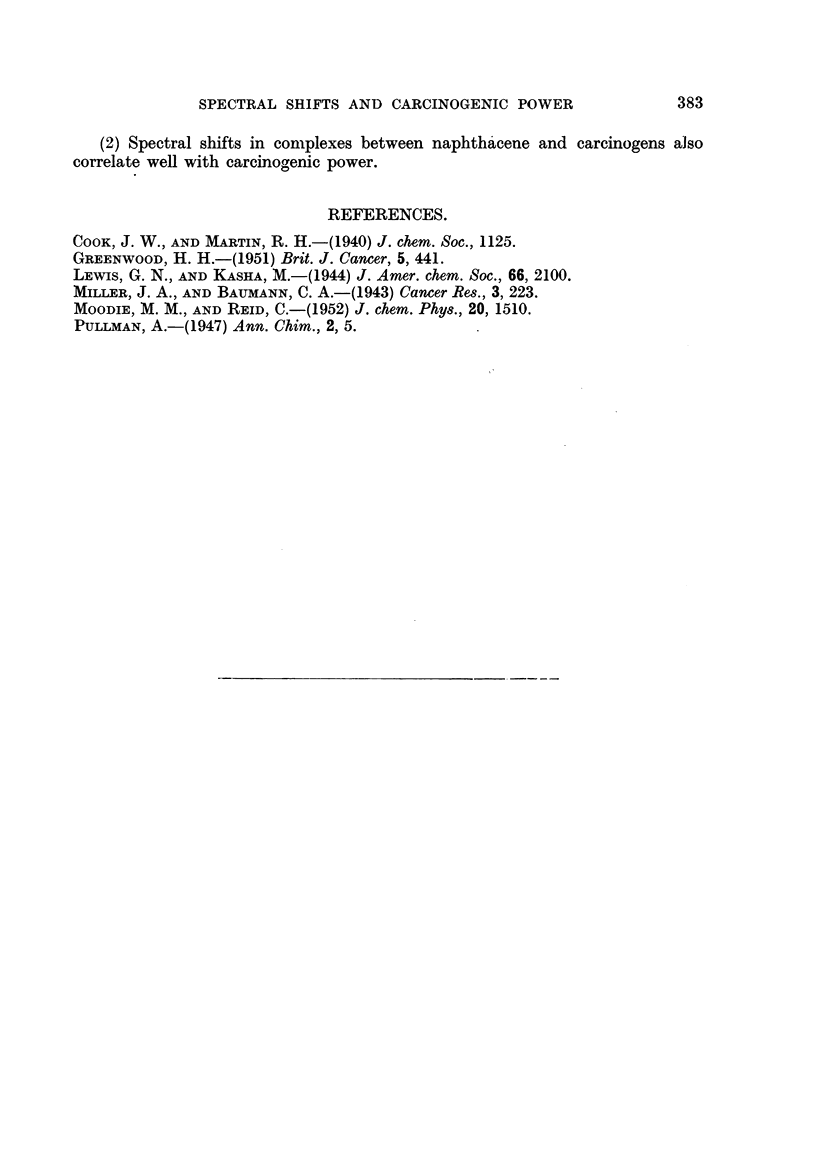# Correlation of Spectral Shifts and Carcinogenic Power

**DOI:** 10.1038/bjc.1954.39

**Published:** 1954-06

**Authors:** M. M. Moodie, C. Reid


### CORRELATION OF SPECTRAL SHIFTS AND CARCINOGENIC POWER

M. M. MOODIE AND C. REID.

*University of British Columbia, Vancouver, B.C., Canada.*

Received for publication April 23, 1954.

MANY attempts have been made to relate the carcinogenic activity of aromatic hydrocarbons to measured and calculated physical properties with very little success. Ease of photoxidation (Cook and Martin, 1940), electronic spectra (Miller and Baumann, 1943), polarizability, free valence at a particular atom, bond order of a particular bond (in so-called K region), (Pullman, 1947), self-polarizability (Greenwood, 1951) are typical examples.

We have examined the visible and ultra-violet spectra in absorption and in emission of a large number of closely related carcinogens and have attempted to correlate them with carcinogenic power. We have also examined various spectral properties depending on the degree of interaction of the carcinogen with other molecules in its vicinity, since the initial step in the chain of events resulting in carcinogenesis must involve some such interaction.

### EXPERIMENTAL.

Precise experimental details are given elsewhere (Lewis and Kasha, 1944; Moodie and Reid, 1952). The emission spectra which yielded most results of interest were obtained by dissolving (or suspending, in the case of the heterogeneous systems examined) the carcinogen in an inert aliphatic medium which could be frozen to a clear transparent glass at the temperature of liquid nitrogen (–190° C.). The samples were frozen in cylindrical tubes immersed in a quartz Dewar vessel and were irradiated with filtered light from a high pressure mercury arc, either the 3100 Å or the 3650 Å group of lines being used. Under these conditions all the molecules examined were strongly luminescent, showing both a short-lived fluorescence (persisting for less than $10^{-8}$ seconds) and long-lived phosphorescence (persisting for several seconds) in different spectral regions. Fluorescence spectra were traced photoelectrically using a Hilger $E_2$ spectrograph equipped with a Scanning Unit. Phosphorescence spectra were photographed using a mechanical phosphorescope as described elsewhere (Lewis and Kasha, 1944; Moodie and Reid, 1952). Both solvents and carcinogens were purified by repeated distillation and chromatography where necessary.

### RESULTS AND DISCUSSION.

(1) No significant correlation could be found between absorption or short-lived spectra and carcinogenic activity, and these spectra, which have already been considered by other workers, will not be discussed. However, some correlations exist between the position of the phosphorescence bands, which lie for the most part in the red region of the spectrum, and carcinogenic power. The results are shown in Table I. In the set of 15 substituted 1,2-benzanthracenes, most of the strong carcinogens have phosphorescence band origins at or below 16,000 $cm^{-1}$ (i.e., above 610 millimicrons) while, in general, the origins of the weak carcinogens' phosphorescence bands lie above 16,400 $cm.^{-1}$. Similarly in the series of 3, 4-benzophenanthrene* the strong carcinogens have phosphorescence band origins below 19,800 $cm.^{-1}$ (i. e., above 505 millimicrons) while the weak carcinogen's phosphorescence has a higher energy.

TABLE I.—*Positions of Emission Band Maxima for the Long-lived Phosphorescence Bands of Some Hydrocarbons and Carcinogenic Power.*

| Hydrocarbon. | Activity. | Origin of phosphorescence band system $cm.^{-1}$. |
|---|---|---|
| 1, 2-benzanthracene | 0 | 16,500 |
| 9, 10-dimethyl-1, 2-benzanthracene | ++++ | 15,500 |
| 10-ethyl-1, 2-benzanthracene | +++ | 16,370 |
| 1'methyl-1, 2-benzanthracene | 0 | 16,980 |
| 2' ,, ,, ,, | 0 | 16,640 |
| 3' ,, ,, ,, | 0 | 16,720 |
| 4' ,, ,, ,, | 0 | 16,670 |
| 3-methyl-1, 2-benzanthracene | + | 16,810 |
| 4 ,, ,, ,, | + | 16,980 |
| 5 ,, ,, ,, | ++ | 16,650 |
| 6 ,, ,, ,, | + | 16,530 |
| 7 ,, ,, ,, | + | 16,580 |
| 8 ,, ,, ,, | + | 16,780 |
| 9 ,, ,, ,, | ++ | 16,260 |
| 10 ,, ,, ,, | +++ | 16,310 |
| 3, 4-benzophenanthrene | + | 19,840 |
| 1-methyl-3, 4-benzophenanthrene | Unknown | 18,940 |
| 2 ,, ,, ,, | ++ | 19,760 |
| 3 ,, ,, ,, | ++ | 19,760 |
| 4 ,, ,, ,, | ++ | 19,720 |
| 5 ,, ,, ,, | +++ | 19,720 |
| 6 ,, ,, ,, | +++ | 18,690 |

This result may be a significant one. The phosphorescence spectra are the result of excitation to the very lowest excited state of the carcinogen molecule, a " triplet " state resulting from the fact that there are two unpaired electrons. Excitation of this state requires only about 45 k.cal., much less energy than is necessary to break bonds in the molecule, and its formation may well be an intermediate step in the reaction with protein. In this connection it should be pointed out that the triplet levels, since they cannot be detected in absorption, the common method of observing spectra, have been much neglected by biologists in their considerations of low barrier reaction paths that may be involved in biochemical reactions.

(2) The second significant correlation comes from heterogeneous system experiments. When suitable pairs of molecules are incorporated in the same system the fluorescence spectrum of the resulting mixture often shows changes due to interaction between the two components (Lewis and Kasha, 1944 ; Moodie and

* 3, 4-benzophenanthrene is the compound called benzo (c) phenanthrene in *Chemical Abstracts* (U.S.A.).

Reid, 1952). In the case of pairs of aromatic hydrocarbons the interaction between two such substances in true solution is very weak indeed at the low concentrations necessary for low temperature fluorescence experiments. Much stronger interactions are often observed if either one of the components is in the form of a microcrystalline suspension while the other is in true solution. Most of the emitted light then appears to come from the surface of the crystals where a layer of the dissolved component is adsorbed. We have found that if a carcinogen is used as the microcrystalline component and the dissolved component is naphthacene, the (fluorescent) emission of the naphthacene is shifted slightly in wave length because of its interaction with the carcinogen. Some of the results are shown in Table II. It will be observed that the smallest shifts in the naphthacene bands occur when the strongest carcinogens are used as the host crystal. There is a moderately good correlation between these shifts and the self-polarizabilities at the position of the substituent calculated by Greenwood (1951), which values themselves correlate better with carcinogenic power than do any other physical constants. The explanation for the observed phenomena must be that the more polarizable the host crystal the more the adsorption process is accompanied by distortion of its orbitals, leaving the orbitals of the adsorbed naphthacene unchanged. Conversely if the host crystal molecule is not polarizable the naphthacene is considerably polarized in the crystal field with corresponding shift of its electronic energy levels.

TABLE II.—*The Shift from its Value in Solution of the Naphthacene Emission Band at* $-190°$ *C. in Suspensions of Various Hydrocarbons compared with the Carcinogenic Activity* (*Pullman,* 1947) *and Self-polarizability Values* (*Greenwood,* 1951), *at the Substituent Position.*

| Component in suspension. | Naphthacene solution band shift. | Activity. | Self-polarizability. |
|---|---|---|---|
| 1, 2-benzanthracene . . . | 603 cm.$^{-1}$ . | 0 . | — |
| 9, 10-dimethyl-1, 2-benzanthracene | 306 ,, . | ++++ . | — |
| 10-ethyl-1, 2-benzanthracene . | 392 ,, . | +++ . | — |
| 1′ methyl-1, 2-benzanthracene . | 561 ,, . | 0 . | 0·439 |
| 2′ ,, ,, ,, . . | 645 ,, . | 0 . | 0·410 |
| 3′ ,, ,, ,, . . | 634 ,, . | 0 . | 0·404 |
| 4′ ,, ,, ,, . . | 498 ,, . | 0 . | 0·439 |
| 3 ,, ,, ,, . . | 624 ,, . | + . | 0·448 |
| 4 ,, ,, ,, . . | 392 ,, . | + . | 0·447 |
| 5 ,, ,, ,, . . | 392 ,, . | ++ . | 0·452 |
| 6 ,, ,, ,, . . | 645 ,, . | + . | 0·409 |
| 7 ,, ,, ,, . . | 686 ,, . | + . | 0·410 |
| 8 ,, ,, ,, . . | 498 ,, . | + . | 0·449 |
| 9 ,, ,, ,, . . | 561 ,, . | ++ . | 0·496 |
| 10 ,, ,, ,, . . | 434 ,, . | +++ . | 0·514 |

For molecules other than the 1,2-benzanthracenes self-polarizabilities were not available to us, but the correlations with biological activity were less convincing.

### SUMMARY.

(1) Within a series of carcinogens with the same basic ring structure carcinogenic power correlates with position of the lowest triplet level (phosphorescence emission spectrum).

(2) Spectral shifts in complexes between naphthacene and carcinogens also correlate well with carcinogenic power.

### REFERENCES.

Cook, J. W., and Martin, R. H.—(1940) *J. chem. Soc.*, 1125.
Greenwood, H. H.—(1951) *Brit. J. Cancer*, **5**, 441.
Lewis, G. N., and Kasha, M.—(1944) *J. Amer. chem. Soc.*, **66**, 2100.
Miller, J. A., and Baumann, C. A.—(1943) *Cancer Res.*, **3**, 223.
Moodie, M. M., and Reid, C.—(1952) *J. chem. Phys.*, **20**, 1510.
Pullman, A.—(1947) *Ann. Chim.*, **2**, 5.